# Personalized Medicine, Disruptive Innovation, and “Trailblazer” Guidelines: Case Study and Theorization of an Unsuccessful Change Effort

**DOI:** 10.1111/1468-0009.12455

**Published:** 2020-05-20

**Authors:** ALEX RUSHFORTH, TRISHA GREENHALGH

**Affiliations:** ^1^ University of Oxford; ^2^ Joint first authors

**Keywords:** disruptive innovation, precision medicine, clinical practice guidelines, sociology of hope, strong structuration theory

## Abstract

**Policy Points**

- For complex reasons, the promise of “precision medicine” based on molecular pathways remains unrealized for many conditions.
- Clinical practice guidelines (theoretically, at least) can act as “trailblazers” to introduce tests and treatments that reflect precision medicine discoveries.
- We describe a detailed case study from the United Kingdom in which such an attempt was (so far) unsuccessful and show how this case provides generalizable lessons.
- Policymakers should be wary of using clinical practice guidelines as the sole, or even the primary, lever for introducing precision medicine.

**Context:**

Precision medicine, which addresses underlying molecular mechanisms of disease, depends on new technologies that measure specific biomarkers, leading (it is anticipated) to more accurate diagnosis, patient stratification, and tailored treatment. These technologies can be disruptive—that is, they make possible, and often require, radical changes to clinical practice and service organization—thereby improving quality, safety, or efficiency of care. Clinical practice guidelines may act as “trailblazers,” introducing and legitimizing novel technologies and practices.

**Methods:**

We describe a case study of an attempt by academic researchers to radically change asthma management in the United Kingdom using a precision medicine biomarker (fractional exhaled nitric oxide, FeNO), measured using a portable breath device. We collected a wide‐ranging data set that included more than 100 documents, 61 interviews, and 150 hours of ethnographic observation, and we analyzed it using technology‐enhanced strong structuration theory (TESST).

**Findings:**

Our study describes a so‐far unsuccessful attempt by academic respiratory medicine researchers to pave the way for a precision medicine approach to asthma using a government‐endorsed national guideline. These researchers considered asthma management, especially in primary care, to be characterized by overdiagnosis and poor tailoring of treatment; engaged a national guideline development body in an effort to fix this problem; and ensured that the guideline required primary care clinicians to use FeNO technology for diagnosis and monitoring. However, clinicians working outside the tertiary referral centers did not accept, or agree to enact, the vision of precision medicine inscribed in the guideline—for multiple professional, operational, and economic reasons.

**Conclusions:**

“Trailblazer” guidelines, in which new technologies are recommended, may succeed as catalysts of change only in a limited way for interested individuals and groups. In the absence of a wider program of professionally led and adequately resourced change efforts, such guidelines will lack meaning, legitimacy, and authority among intended users and may be strongly resisted.

High‐technology medical research is not without its critics. Sociologists talk of “regimes of hope”—the promise of medical advances—in tension with “regimes of truth”—the actual performance of these advances.^1^ This is particularly true for the field known as “precision,” “personalized,” “stratified,” or “P4” (predictive, preventive, personalized, participatory) medicine, in which attention to the molecular pathways and genetic abnormalities of diseases and predisease states is said to offer more accurate diagnosis, firmer prognosis, and more tailored treatments.^2^, ^3^

A critical literature in sociology and philosophy of science depicts precision medicine as hyperbolic and reductionist: a seductive idea, based on flawed assumptions about how closely specific molecular processes link to illness and its prevention and treatment.^4^, ^5^, ^6^, ^7^, ^8^ Precision medicine, say critics, misrepresents the past as offering a crude, primitive version of medicine (since, in reality, doctors have always sought to tailor management to the individual) and that individualization based on molecular “targets” is a retrograde step compared with individualization based on skilled history taking, full clinical examination, and conventional tests as appropriate.^4^, ^6^, ^8^, ^9^

While such critiques are sometimes insightful, they do not always acknowledge the substantial improvements in survival that a molecular or genetically targeted approach has brought in diseases such as HIV,^10^ hypercholesterolemia,^11^ chronic myeloid leukemia,^12^ and breast cancer^13^—though in all these examples, not all patients possess the relevant biomarker. Indeed, when the scientific gaze is directed to “personalized” (ie, molecularly derived) solutions, new forms of health inequality may be created, since patients whose illness lacks the target molecule of interest will be excluded.^14^ However, the search for new molecular pathways may identify additional biomarkers and generate new therapies for previously untreatable forms of a disease, as has happened recently with what was previously known as triple‐negative breast cancer.^15^

This brief introduction reveals two competing philosophical perspectives on personalized or precision medicine. The *essentialist* perspective depicts thousands of undiscovered biomarkers that, once identified, will allow us to rewrite the textbooks of what disease is and how it should be monitored and treated, with dramatic benefits accruing to both patients and health care systems. The *performative* perspective, which takes its name from actor‐network theory and the notion that words “do things,”^16^ holds that expectations (ie, the “hype” of high‐technology, molecularly focused medical research) do not merely describe a hoped‐for future. They also help to bring that future into being—for example, by mobilizing the interests of various actors (notably innovation and regulatory networks), defining roles, and creating mutually binding obligations.^4^, ^5^, ^6^ The former position sees a single future whose advent awaits the march of science; the latter implies other, perhaps better, futures from which the prevailing scientific paradigm is distracting us.

## Precision Medicine as Disruptive Innovation?

The technologies of precision medicine (new diagnostic tests, new drugs) make possible new service models and care pathways. Tests that previously required a secondary care setting (eg, a laboratory) can now be done using point‐of‐care technologies in primary care or even by the patient at home, with or without remote transmission of data to the clinician. These technologies are sometimes classified as *disruptive innovations* because—and to the extent that—they imply new roles for both patients and clinicians and new organizational and interorganizational routines.^17^ Although the term is popular with those seeking to implement radical change, many so‐called disruptive innovations in the commercial sector in reality failed to disrupt existing markets.^18^

In the health care setting, and in the context of escalating costs and demographic shifts toward an aging, multimorbid population, the term *disruptive innovation* is increasingly used to depict a hoped‐for situation in which “complicated, expensive products and services are eventually converted into simple, affordable ones.”^19^ ^(p1329)^ In 2018, the newly appointed UK secretary of state for health, Matt Hancock, quickly aligned himself with technological entrepreneurs whose products he believed offered potential streamlining of care pathways and thus efficiency savings;^20^ he vowed to “stand up to vested interests” in the National Health Service (NHS).^21^

The assumption that disruptive technologies can drive major changes to services is an example of the largely discredited theory of technological determinism.^22^ Technologies do not emerge in a vacuum; they are the products of particular social and political forces, and it is these wider forces, not merely the technologies themselves, that shape and constrain the direction of change. The extent to which Mr. Hancock's favored businesses “disrupt” the NHS, for example, will depend as much on the innovation and regulatory networks he helps to build as on the inherent capabilities of the technologies themselves.

Organizational scholars Fitzgerald and McDermott analyzed a number of UK‐based initiatives to implement “disruptive” organizational changes in health care.^23^ They concluded that although the *idea* of disruptive innovation has much rhetorical appeal, as a policy it has tended to fail because enduring change in complex health systems—in the rare cases that it happens at all—tends to be incremental and organic and to occur with a minimum of disruption.

The literature on disruptive innovation thus also reflects a tension between, on the one hand, the essentialist position that innovations have inherent transformative properties and are capable of driving a revolution in health care services and systems and, on the other hand, the performative position that these technologies are as much a product of transformative forces as their cause, and hence will become mainstream only if social, political, and economic forces play out in their favor.

## Clinical Practice Guidelines as an Instrument of Change

Clinical practice guidelines summarize evidence‐based recommendations; they also standardize and rationalize care, partly by producing “action at a distance” (ie, recommending, if not requiring, local clinical decisions to follow standards set centrally).^24^ Few contemporary scholars take a purely essentialist view of guidelines, viewing them as neutral conduits of scientific truth, since even when efforts are made to reduce bias, the guideline development process requires input from multiple stakeholders, who must, individually and collectively, make subjective judgments. It also requires a source of funding.^25^, ^26^

Critical social scientists go further, viewing guidelines in overtly performative terms as instruments of power—not least because they are visible statements of professional consensus. In such accounts, guideline development is presented as an inherently political process in which different professional and countervailing managerial groups struggle to exert and defend their jurisdiction and influence.^27^, ^28^, ^29^, ^30^ Research in the performative tradition has addressed how guidelines gain authority, legitimacy, and meaning in shaping the value of health technologies.^31^, ^32^, ^33^ Some studies have drawn on the sociology of expectations to highlight both the “gatekeeping” role of guidelines, whereby guidelines curtail premature adoption of medical innovations that do not yet have strong evidence of efficacy,^34^ and the “trailblazing” role of guidelines in inscribing future aspirations for emerging technologies, setting the stage for them to become legitimized in the future.^5^

Boenink, for example, describes how a clinical practice guideline for Alzheimer's disease embraced both a regime of truth, explicitly advising *against* current use of a classification system based on molecular biomarkers, and a regime of hope, depicting biomarker‐based reclassification of Alzheimer's disease as on the horizon.^5^ The guideline thus achieved a subtle performance: eschewing precision medicine for the present while at the same time embracing it for the future.

The philosophical assumptions underpinning essentialist and performative perspectives correspond respectively to two contrasting meanings of the term *translation*. In conventional, essentialist medical parlance, knowledge translation refers to efforts to implement research findings that are viewed, more or less, as generalizable truths.^35^ The sociology of translation has a different, performative meaning—namely, efforts to stabilize a particular alignment of human and technological actors in a complex system.^36^ As Callon observed, his kind of translation is seen as occurring in four stages: problematization (defining a problem for which a particular technology is a solution), interessement (getting others to accept this problem‐solution), enrollment (defining key roles and practices in the network), and mobilization (engaging others in fulfilling the roles, undertaking the practices, and linking with others).^36^ Such attempts are typically conflict ridden, and people may mobilize to enact and stabilize an “antiprogram” in which the technology is used differently, or not at all. Resistance to technologies may be studied in terms of the program‐antiprogram dynamic.^37^, ^38^

The rest of this paper describes an empirical case study, analyzed through two contrasting analytic lenses—essentialist versus performative—on the linked themes of precision medicine, disruptive innovation, and clinical practice guidelines. Our overarching research question was, to what extent did the discovery of a new molecular biomarker for asthma drive changes in classification and management of the disease, and what role did a new clinical guideline that embraced this biomarker play in this process?

The remainder of this paper is structured as follows: First, we provide the background to our case study, including the context for precision medicine and guideline development in the UK, and describe our sampling frame and data collection methods. Next, we introduce our theoretical framework, based on Greenhalgh and Stones' technology‐enhanced strong structuration theory (TESST). We then describe the data analysis methods we used to build a rich, multilevel case study. In the results section, we offer an analysis of how the case played out at macro (institutional), meso (organizational), and micro (clinical encounter and technology in use) levels. In the discussion, we reflect on the limited success to date of efforts to achieve a “precision medicine” approach to asthma management and draw out wider lessons about precision medicine technologies more generally.

## Methods

Inspired by previous sociological accounts of translational research,^5^, ^39^, ^40^ we chose to trade breadth for depth by using a single in‐depth case study. The case concerns an attempt by academic respiratory medicine researchers to introduce a new classification scheme and management approach for asthma. It was based on a molecular biomarker that reflected the concentration of eosinophils in lung tissue, measured using a new diagnostic technology known as fractional exhaled nitric oxide (FeNO), whose features are summarized in Box 1. Eosinophils, a kind of white blood cell, usually proliferate in an allergic reaction and produce nitric oxide, so a high FeNO reading, referred to as type II high or eosinophilic airway inflammation, implies that the underlying pathological process is allergic. A low FeNO reading indicates a nonallergic process. This research emerged in the context of a growing belief that doctors were overtreating some forms of lung disease with steroids. Steroids have powerful anti‐inflammatory properties and can be lifesaving in asthma, but they also have serious side effects. Patients with no evidence of lung eosinophilia could, it was argued, safely be spared steroids.^41^, ^42^ Accordingly, there was a molecular basis, a bedside test, and the potential to refine diagnostic categories, personalize treatment, and reduce harm. Recommendation to use the FeNO test was included in a new national guideline, known as NG80, which (after considerable controversy, described later in this article) was published in 2017.^43^

Box 1 **Material Properties of the FeNO Test**

- Point‐of‐care test measures exhaled nitric oxide as a biomarker for eosinophilic airway inflammation.
- Three testing device kits, made by different manufacturers, have been evaluated and endorsed as clinically and cost effective by NICE.
- Devices are battery powered, handheld, and certified to meet environmental health and safety standards.
- It takes approximately one minute to display result (in parts per billion) on device screens.
- Price of devices varies from £1,794 to £2,540, plus £4.93 to £9.35 per test for the sensor and filter.
- Devices have limited numbers of tests built in (quantity not publicly stated). When limit is reached, a new device must be purchased.

### The Context: UK Translational Medicine

The UK has a well‐established infrastructure for translational medicine. Partnerships between research institutions and the publicly funded NHS receive more than £1 billion ($1.3 billion) per year of government funding, much of it from the National Institute for Health Research (NIHR). These academic–NHS partnerships include 18 biomedical research centers (BRCs), located in centers of excellence, whose remit is to make world‐leading discoveries in basic science, ensure that these are followed through to generate benefits to patients and improve service efficiency, and train researchers.^44^ Our chosen case study is paradigmatic of how BRCs’ remit depicts translational research as progressing from bench to bedside as technologies and drugs emerge from laboratory studies, gain regulatory approvals, and become enshrined in national guidelines.^45^

The UK also has a strong tradition of government support for evidence‐based clinical practice and policy. The National Institute of Health and Clinical Excellence (NICE) is a world‐renowned producer of technology appraisals and clinical practice guidelines; NHS organizations are expected (though not always required) to follow its recommendations. NICE views its remit as publishing guidelines that are, in the words of its former director, “almost aspirational in nature”—that is, driving infrastructure changes that enable the NHS to deliver the highest standards of care.^46^ This well‐intentioned strategy has not always gone down well with professional groups. NICE has been challenged for producing guidelines that seek to reshape primary care in the image of hospital‐based specialist care when the populations served, patterns of disease, and available technologies are very different^47^, ^48^, ^49^—a critique that lies at the heart of the case study we describe in this paper.

### Management and Governance of the Study

This study was part of the Partnerships for Health, Wealth, and Innovation (PHWI) research theme within the NIHR Oxford BRC, which seeks to explore contemporary translational medicine from an interdisciplinary perspective.^44^ PHWI takes as its main focus of study the numerous clinical research studies funded by the Oxford BRC. The study reported here is based in the respiratory medicine research theme. PHWI has an external advisory group with a lay chair and representation from academia, service providers, industry, patients, and the lay public. Ethical approval was provided by the University of Oxford Inter‐Divisional Research Ethics Committee (June 9, 2017, reference no. R51801/RE001). In addition, the patient‐facing clinical elements were covered by NHS Research Ethics Committee approval (July 21, 2015, reference no. 15/SC/0404).

### Data Sources and Collection Methods

Data sources are summarized in Table 1. We undertook an ethnographic case study consisting of collection and analysis of 60 documents, 14 “elite” narrative interviews with BRC theme leads (all senior professors), 47 semistructured interviews with a maximum variety sample of stakeholders (Table 2), and 150 hours of ethnographic observation. This rich data set was chosen to provide first‐order descriptive insights and higher‐order categorizations for considering the macro‐, meso‐, and microlevel elements of a complex case study of institutional change (and, as it turned out, resistance to change).

**Table 1:** Overview of Data Structure and First‐ and Second‐Order Interpretations

Research Focus	Type and Nature of Data	First‐Order Interpretations	Second‐Order Interpretations
Macrolevel study of wider context for technology‐driven change in UK health policy	National policy documents and guidelines (*n* = 37); News articles and press releases (*n* = 22); Oxford NIHR BRC grant application (540 pages) Total documents = 60 “Elite” interviews with Oxford NIHR BRC theme leads (*n* = 14) on precision medicine research and technological change in the NHS	Historical and policy drivers for the move to FeNO testing and precision medicine in secondary and primary care Development and introduction of NICE Asthma Diagnostic Guideline NG80	External social structures such as Clinical and academic discourses on precision medicine (including prevailing epistemic models of what asthma *is*; professional standards and definitions of excellence, such as what is evidence‐based asthma care) Political, regulatory, and economic context of NICE guideline development How this external context had been (and was being) shaped by the actions of individuals and groups
Mesolevel study of organizational change efforts	Ethnographic observation of respiratory clinical research team; included weekly research meetings, seminars, multidisciplinary team meetings, outpatient clinics, shadowing research (150 hours) Audiotaped semistructured interviews with stakeholders in NICE NG80 development and implementation (*n* = 47; see Table 2)	Observations and actors’ descriptions of introducing FeNO in secondary care (hospital outpatient clinic and research team) Descriptions of introducing (or choosing not to introduce) FeNO in primary care (GP surgeries)	“Scripts” held by staff about how they and others do or should behave in organizational settings and how these scripts are changing over time Assumptions built into organizational routines and logics about, for example, capability of users, how professionals diagnose people with asthma, the nature of clinical work and routines, and how all these interact
Microlevel study of individual actors’ perspectives and assumptions inscribed in the FeNO technology	47 semistructured stakeholder interviews (as above) Examination of diagrams and graphs in NICE's NG80 guideline and FeNO technology appraisal review	Accounts of what individual actors “knew” about NG80 and FeNO technology (including incomplete or inaccurate beliefs) Diagrammatic depictions of “the way things are,” as portrayed in NG80 Material properties and affordances of the FeNO device	How human actors (eg, researchers, GPs) internalize and interpret prevailing professional discourses and the political, economic, and technological context for the introduction of NG80 Assumptions built into NG80 about, for example, capability of users, how health care professionals diagnose people with asthma, the nature of clinical work and routines, and how all these interact How the material properties of FeNO technology shape and constrain asthma diagnosis and management in secondary and primary care

**Table 2:** Breakdown of Interviews by Actor Type

Type of Actor	No. of Interviewees
NICE Guideline Development and Technology Appraisal Committee members	5
Research scientists	13
Primary care professionals	14
Commercial representatives	7
British Thoracic Society and Scottish Intercollegiate Guidelines Network (BTS/SIGN) members	5
Research charity policy and patient representatives	3
NIHR Oxford Biomedical Research Centre theme leads	14
Total	61

The main goal of our macro‐ (societal) level data collection was to study how the NICE NG80 guideline came about, and within this process, how FeNO testing came to be constructed as a solution to a wider set of concerns surrounding asthma care.^36^ Documents included the history of the guideline (including minutes of meetings, draft versions, stakeholder lists, consultation comments, and responses from NICE) and feedback (much of it critical, from professional organizations) submitted to NICE at various stages of the consultation process. We also studied press releases and press articles, clinical guidelines produced by other groups,^50^, ^51^ and scientific literature and reports relating to FeNO testing.

At the meso (organizational) level, our data collection was oriented to informing an analysis of organizations’ efforts to “translate” clinical practice around the NG80 recommendation for FeNO testing. We shadowed and interviewed staff in the respiratory medicine research theme and observed how FeNO testing was (largely successfully) routinized in this academic secondary care setting. We also sought to explore, through interviews with primary care professionals—who were less familiar with, and less keen on, FeNO—the “fit” between NG80 recommendations and existing organizational routines and scripts for managing asthma. NICE's consultation documents were useful for identifying organizations that had commented on the workability of FeNO testing; we contacted such organizations and invited them to nominate representatives for interviews.

In our microlevel data set, we sought to capture the perspectives of individuals, especially health professionals undertaking asthma care. Ethnographic observation allowed us to study the technology in use, including how actors interacted with material features of FeNO‐testing devices, and their perceptions about its ease of use and reliability. We incorporated a secondary analysis of ethnographic field notes taken in primary‐care‐based asthma clinics from our previous research.^52^

### Theoretical Approach: Technology‐Enhanced Strong Structuration Theory

Strong structuration theory (SST) was developed by Stones as an empirically focused adaptation of Giddens's structuration theory^53^ and later extended for the study of technology‐driven change by Greenhalgh and Stones,^54^ who drew eclectically on actor‐network theory (ANT).^36^, ^55^

SST is concerned with the relationship between human actors and social structures. It considers both *external* structures (such things as societal and professional norms, laws, and cultural expectations) and how those structures are *internalized* by people as knowledge, experience, morals, and patterns of learned behavior. In any social situation, human actors draw on both their habitus (ie, their internal dispositions, beliefs, values, norms, and so on, acquired over the years) and on their assessment of the here‐and‐now strategic terrain and how they are expected to act within it. Their action will have short‐ and long‐term consequences; it sometimes reinforces, and may ultimately change, social structures. SST views human actors as connected in networks that are constantly evolving. Each has a position in the network (eg, as a doctor or a patient) that comes with socially sanctioned and expected ways of behaving (what Stones calls a position‐practice^53^).

Drawing on ANT, technology‐enhanced SST (TESST) considers technologies as part of these dynamic, evolving networks. But unlike ANT, it allows the researcher to analyze in detail the internal justifications offered by human actors. Furthermore, TESST views humans as fundamentally different from technologies—for example, the former are moral beings with the capacity for imagination, emotion, and reflexivity, while the latter are not. But TESST accepts that technologies, too, internalize and enact—in a limited, nonhuman way—institutional rules, since their design reflects what is socially expected, what is organizationally permitted, and what is considered good professional practice. As Bowker and Star remind us, technologies contain “frozen organisational and policy discourse.”^56^ ^(p189)^

The networks envisaged by TESST are inherently unstable, but they can be stabilized to some extent when people, technologies, standards, procedures, training, incentives, and so on are aligned—Callon's sociology of translation referred to earlier.^36^ The introduction of precision medicine for asthma via the NG80 guideline can be seen as an attempt at translation.

TESST thus offers the potential for rich theorization of the development, adoption, and nonadoption of the NICE NG80 asthma guideline along with its “disruptive technology” (FeNO testing). It allows us to explore actors’ knowledgeability of contemporary professional practice, the prevailing pro‐innovation policy context, and the practical and economic realities of the UK NHS. It also enables us to surface and theorize the unintended consequences of an attempt to “shake up” asthma care among professional groups in the adopting system.

### Data Management and Analysis

Our 47 semistructured interviews lasted between 30 and 105 minutes. They were audio‐recorded with consent and professionally transcribed. The 14 elite interviews lasted between 30 and 60 minutes. These were not audiotaped, but detailed contemporaneous notes were made. Together with documents and field notes, these free text data were imported into the NVivo software system and an initial thematic analysis using open coding was undertaken to gain familiarity with the data. We then applied a higher‐order analytic lens, based on TESST, as follows.

Following a methodology developed previously,^38^, ^54^ we focused primarily on what Stones calls “conjunctures”—that is, small‐scale social situations, such as a clinical consultation—in which human actors draw on their internal dispositions and their assessment of the strategic terrain to first decide how to act and then execute that action, supported or constrained by available technologies. While a conjuncture is by definition a microlevel phenomenon, it provides a window for the exploration of meso and macro social structures via a set of indirect questions.

At the macro level, we asked: What is the prevailing context within which asthma care is undertaken in the NHS and the NICE asthma guideline NG80 has emerged? What realignment of the sociotechnical network (people, technologies, position‐practices, relationships) is implied by NG80, and how have its architects attempted to achieve this realignment? To what extent has stability of the network been achieved and why?

At the meso level, we asked: What are the implications of the NG80 guideline generally and FeNO testing in particular on organizational routines and practices in secondary and primary care?

At the micro level, we asked: What are the general dispositions of potential adopters of the NG80 guideline and the FeNO‐testing technology, and what do these individuals know (perhaps imperfectly) about relevant external social structures? How does the guideline and the practice it recommends fit with their perceptions of professional standards of excellence? What do they think other actors think? What external social structures are inscribed in the NG80 guideline and the FeNO technology? How do the technology's material features play out in the clinical encounter and how do they shape and constrain the changes in clinical practice envisaged by the guideline's architects? Finally, what are the consequences of these actions both short and long term?

Through reflection, discussion, and a constant comparative approach, we were able to develop an overall picture of the case and add depth and detail to it as more data were incorporated. Our findings are presented as a composite narrative organized across three sections of macro, meso, and micro analysis followed by a discussion section that synthesizes and reflects on these findings.

## Results

We present our findings as macro‐level (the strategic context), meso‐level (organizational) and micro‐level (individual clinicians and clinical encounters).

### Macrolevel Findings: The Strategic Context for NG80 and FeNO Testing

As noted in the Methods section, the NICE asthma guideline emerged against a backdrop of wider debates over perceived deficiencies in NHS asthma care, particularly primary care,^57^ especially the imprecise targeting of potentially harmful steroid drugs.^41^, ^42^ Despite a fall in asthma deaths and hospital admissions,^58^ concerns about deficiencies in care remained. One study claimed that up to 30% of patients given the diagnostic label of asthma did not have the condition when “objective” tests were used, and hence were being exposed to steroids unnecessarily.^59^

The positioning (by a somewhat ad hoc coalition of academically oriented respiratory physicians) of FeNO testing as a solution to these issues is, in an essentialist analysis, a straightforward response to the discovery of a new scientific “fact.” But in a performative analysis, this positioning is an example of problematization—Callon's first stage of translation.^36^ In 2012 the UK Department of Health commissioned NICE to produce its first asthma guideline, emphasizing “objective testing” in diagnosis.^60^ The draft guideline, known as NG80, appeared in 2015; the final version was published in November 2017. (Earlier drafts are accessible from the NG80 page on the NICE website at www.nice.org.uk.^43^)

From early on, the NG80 guideline attracted controversy. NICE's initial press release cited the contested “30% overdiagnosis” claim.^59^ This was picked up by national news sources, leading to criticism from various stakeholders, notably the UK Royal College of General Practitioners and the Primary Care Respiratory Society (a GP professional interest group). Particularly contentious was NG80's recommendation, avowedly based on “strong” research evidence,^43^ that patients should not be given the diagnosis of asthma in primary care without FeNO testing. This was considered by both proponents and skeptics as a gestalt shift from existing approaches based on holistic clinical assessment comprising history, clinical examination, and peak expiratory flow rate (a simple test of puff using a cheap spring‐loaded device), along with a pragmatic trial of a bronchodilator drug.

The status of FeNO as a precision medicine test was disputed from the outset. Notwithstanding the essentialist claim to the existence of “strong” evidence for the FeNO diagnostic test, some clinicians felt it was not even as good as existing tests (for example, the British Medical Association had declared in 2015 that FeNO “was not yet fully validated in diagnosing asthma”^61^). A performative interpretation depicts the champions of precision respiratory medicine, who tended to be very critical of existing asthma guidelines,^51^ constructing a promissory vision: a time in the not‐too‐distant future when asthma patients would no longer be classified along broad clinical categories such as mild, moderate, and severe (or “difficult to control”) on the basis of peak expiratory flow readings, but assigned to a more precise diagnostic category based on biological, lifestyle, and environmental markers of which FeNO would be one, but not the only, element.^41^, ^62^

The NG80 guideline did not, in its first iteration, position FeNO as a state‐of‐the‐art precision medicine test. Rather, the test was billed as an important “objective” test for detecting lung inflammation and thus ruling in or out mild or moderate asthma (though not, importantly, severe asthma). NG80 thus embraced both a regime of truth—that FeNO can already be used as an “objective” test for asthma—and a regime of hope—that FeNO represents a new direction of travel toward precision medicine for primary care asthma management. As one member put it:

So I think that's sort of— That's more looking to the future. And obviously the original diagnostic guideline was done a while ago. It has been updated, but all the time, more evidence is coming out to suggest that FeNO is helpful at predicting response to inhaled steroids. But that's come out since the guidelines closed, so for the next iteration we would imagine we might get that in there.

—NICE NG80 guideline development group member 1

Precision medicine enthusiasts from academia and industry expressed disappointment in NICE for “slowing down” progress toward a precision approach to asthma. The delay, they felt, was due to NICE's bureaucratic procedures and the low status of basic scientific knowledge (compared to empirical findings from randomized trials) in the guideline development process, as evidenced in this clinical researcher interview:

Respondent: I think it's sad they [NICE] don't want to develop the biological description [of FeNO] more in the guidelines. I think they should….

Interviewer: Why would guidelines not take on this biological definition of asthma?

Respondent: It's a highly conservative business. It starts with 19th‐century medicine…. I guess they want to take one step at a time. It will slow down the process. But it's the way it's always worked with guidelines…. Basically it's a lack of fundamental biological and physiological knowledge. They don't know about the underlying biology. That's why they decided this.

As this exchange illustrates, while academic interviewees were frustrated with the pace of travel, they appeared comfortable with its general direction and felt that the precision medicine vision (eg, use of FeNO and other biomarker‐based testing to stratify patients into different care pathways) would eventually be realized through further iterations of the guideline. Some emphasized the symbolic significance of including the FeNO test in the current guideline:

NICE have wanted to get a measure in that was objective, easy to obtain, and was in the area of precision medicine. I think that sends the signal. That that's allowed in a guideline—I think that is a huge step forward. Sometimes somebody needs to push a little bit harder. Whether people will think it's correct to start that way or not doesn't really matter. It will bring attention.

—Professor of respiratory medicine

Respiratory medicine academics, then, viewed the NG80 guideline as a vehicle to get FeNO testing through the door of primary care, thus building familiarity, driving some reengineering of pathways and processes, and preparing the ground for a more radical transformation of asthma care in the future. NICE guideline development group members also portrayed FeNO testing in essentialist terms as an instrument of progress, since it and other “objective” tests would enable asthma care to “catch up” with how other medical conditions are managed (heart failure diagnosis and monitoring, for example, is now based on brain naturietic peptide, BNP). Many depicted asthma management as being on a pathway toward a more “modern” (ie, precise, data‐driven) era and were confident in the bureaucratic stability of NICE to capture this progress in future iterations of the guideline.

[A]dding in an additional test, you're absolutely right, it's starting to move towards precision medicine. So, we know that asthma is more of a syndrome than single diagnosis. There are lots of different causes of having inflammation of the lungs and intermittent wheeze, and intermittent obstruction of the airways. And by measuring the FeNO, the nitric oxide level, we're starting to understand that in that person sitting in front of us, what's driving symptoms.

—NICE guideline development group member 1

Few interviewees from primary care, however, were comfortable with this direction of travel. They, by and large, rejected the “fact” of FeNO as unequivocally representing scientific progress. Rather, they saw developments in more performative terms: they believed the NICE guideline development group had allowed academic precision medicine enthusiasts to use the current guideline to introduce FeNO prematurely as a functional diagnostic and monitoring test for asthma until such time as they could legitimately embrace new evidence about its role as a precision marker. The guideline was viewed as an unwelcome attempt to impose an unnecessary, costly, and impractical change in primary care practice (“enrollment” in Callon's translational process^36^). Notably, at the time NG80 was first introduced, few primary care settings possessed FeNO‐testing kits (which GPs were expected to pay for), and the asthma component of the pay‐for‐performance system for GP remuneration (Quality Outcomes Framework, QOF) did not expect or reward the use of the FeNO test.^63^ It is also noteworthy that the Royal College of General Practitioners, in an unusual act of resistance, refused to endorse the implementation guidance and quality standards that NICE produced alongside the asthma guideline.^64^

Highly relevant to the primary care side of this story is that a trusted and widely used asthma guideline had been produced by the British Thoracic Society (BTS, a network of respiratory physicians) in 1990^50^ and was updated regularly in collaboration with the Scottish Intercollegiate Guidelines Network (SIGN), which represented medical and nursing Royal Colleges.^51^ Whereas almost all other chronic conditions had been managed for years using NICE guidelines (the first NICE guideline for type 2 diabetes, for example, was published in 2002), clinical practice guidelines for asthma had long been “professionally owned” and NICE, a government organization, was seen as coming late, and uninvited, to the party.

In 2015, in response to protests to the draft NG80 guideline from primary care stakeholders, among others, the Department of Health instructed NICE to withdraw it temporarily, pending a feasibility study of FeNO in selected general practices—a unique move in NICE's 17‐year history. The final version of the guideline met with negative editorials in the professional press.^65^ NICE guideline group members responded that they accepted some of the feasibility (though not the validity) issues relating to FeNO testing in NHS primary care and acknowledged that it would take time for the guideline to be adopted in full; in the meantime, they recommended continuing use of the BTS‐SIGN guideline.^66^

All this was occurring in the context of a long period of public‐sector austerity, in which NHS organizations were expected to generate efficiency savings to weather year‐on‐year reductions in budgets^67^ and growing staff shortages in some parts of the NHS.^68^ While precision medicine enthusiasts held the essentialist view that FeNO, and precision medicine more generally, would lead to efficiency savings through better targeting of resources, GPs viewed the picture as more nuanced and did not view such savings as inevitable.

### Mesolevel Findings: FeNO Testing and Organizational Routines

Examples of asthma management routines in secondary and primary care are shown in Boxes 2 and 3, respectively. These have been selected to illustrate key themes in the data set and have been slightly fictionalized to protect identities.

Box 2 **Use of the FeNO Test in a Tertiarary Care Setting: Ethnographic field notes**

In a multidisciplinary team meeting prior to the afternoon outpatient clinic, a consultant reads through the case notes of a patient who will be returning for a second appointment. He summarizes: “This is a young woman who is a student at the university. The patient has biomarker low asthma—her FeNO is low. But asthma isn't the cause of her current problems. She's on huge doses of steroids. There is very good evidence for the theory of steroids causing infections. It's a real failure of symptom‐based management. If we had FeNO in primary care it would nip this kind of thing in the bud.”

Later, the patient arrives for her clinical appointment, accompanied by her father. Following the patient's account of fluctuation in her asthma since moving to college, the consultant moves on to performing a FeNO test with the patient. “For this you need to hear a constant noise. Ready? Breathe in. Go!”

The consultant instructs the young woman, reading the dial on screen and listening to the sound of the device. “More! Lower! Keep it there, too much, too much, keep in the middle!”

The dial has to stay in the middle of the screen for a full 10 seconds for the device to give a successful reading. This time it fails. “Don't worry, we can try again. Okay, breathe in and … *go!*”

This time the machine makes a pleasing beep. It has worked.

While patient and doctor wait for the score to load onto the device screen, conversation continues. The young woman's father, himself a health professional, asks a number of questions of the consultant, who is a well‐known name in the field of severe asthma research. The father mentions that he is reassured that the consultant also finds the young woman's case to be complex. The consultant states, “I'm using FeNO a lot. It helps break down the complexity.”

During this conversation, the consultant checks the FeNO device screen and writes the score by hand onto a paper clinical note inside the patient's medical record.

Turning back to the patient, the consultant says: “You look well. Keep on your medications and we'll see you in a few months. And next time we'll make sure you do the works with our nurses.”

Patient and father exit. At the end of the day's clinic, the consultant dictates a letter to the patient's GP. In it, he states “The patient is non‐eosinophilic and therefore we don't think exacerbations should be treated via prednisolone [oral steroids].”

Box 3 **Asthma Consultation in Primary Care without FeNO Testing: ethnographic field notes**

The first patient had come for an asthma review. When the patient came in, JH (nurse) had his electronic record open to the “Journal” page and said, “Hello, have you come for an asthma check?” The patient confirmed and said he had run out of inhalers a few days ago. JH replied, “We'll get you some more.” She looked at his current list of medications and asked if he was still using the blue (reliever) and brown (preventer) inhalers, and went on to explain the importance of taking the two together. She suggested the patient might want to try a combined inhaler, and after checking his current medications said, “You're on a hefty dose of the brown one. You're on the strongest dose.” The patient nodded but said very little. JH asked to check his peak flow and did so three times, checking his technique and saying things like, “You're not doing badly.” After the final attempt she did not tell the patient what the reading was but turned to the record and added the READ code “peak flow rate” and entered the value. She then turned back to the patient and asked to listen to his chest and told him he was “sounding pretty good.”

While the patient dressed, JH retrieved a box containing placebo inhalers and spacer devices. She ran through the different types of inhalers, including a dry inhaler (Seretide), which she suggested the patient might like to try. She explained that he would only need to take it twice a day, in the morning and at night, and after demonstrating how to use it she asked him to try. The patient said it was “okay,” to which JH responded, “Great, why don't you give it a whirl, see how you get on,” and if he liked it she would add it to his repeat prescriptions. JH then turned to the record and added the next recall appointment for the patient. She also added the Seretide to his current medications.

After the patient left, JH opened the asthma template, where she recorded the patient's smoking status and then clicked on “asthma review.” This opened a new screen and she added the READ code “asthma annual review” and in the comment box wrote, “Not feeling well at present, just getting over cold. Run out of inhalers, pf [peak flow] down but o/e chest clear. Discussed inhalers to try Seretide 500 accuhaler and review in 6 weeks.”

*—*From a previous ethnographic study in UK general practice^52^

The vignette in Box 2 is an example of what SST calls a conjuncture—a microlevel social interaction from which we can glean insights about the internal structures of actors and the macro social and technical structures that shape and constrain human actors’ values, attitudes, and behaviors, and how these macro structures are reflected in mesolevel organizational scripts and routines. The example illustrates, for example, why FeNO testing is already well embedded in the context of this tertiary referral clinic, where it is expected that complex decisions will need to be made about difficult cases. There is a prevailing culture of innovation and horizon‐scanning for new research discoveries. Several FeNO‐testing devices (likely purchased from research budgets or other non‐NHS money) are available within the clinic, and one is switched on and ready to use at the consultant's desk during patient appointments; another is available for use by the nurses. “The works” in this case study refers to the full respiratory workup that is routinely performed in this clinic by the team of nurses, including FeNO but also spirometry, peak flow, blood eosinophil count tests, and oxygen saturation. The consultant can use the clinical record to note a need for FeNO and other forms of testing at the next visit, which the nurses will then carry out.

**Figure 1:**
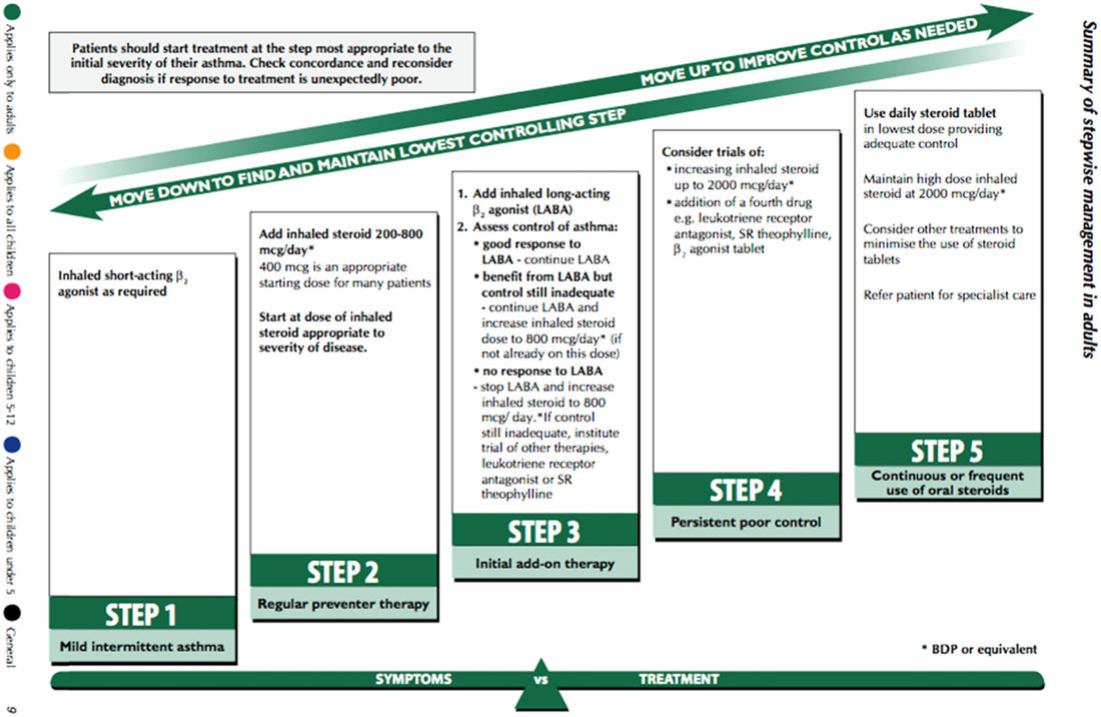
Diagram From BTS‐SIGN Asthma Guideline Showing the Step‐Up, Step‐Down Treatment Ladder^a^ ^a^British Thoracic Society; Scottish Intercollegiate Guidelines Network (2014).^51^ [Color figure can be viewed at wileyonlinelibrary.com]

The situation in primary care is very different. As the vignette in Box 3 (based on an earlier empirical study by our team^52^) illustrates, asthma care has become largely a protocol‐driven and nurse‐led activity, using a structured computer template based on the BTS‐SIGN guideline.^52^ Figure 1 shows a central image from the guideline that conveys its pragmatic focus, with a “step‐up, step‐down” treatment ladder and clear instructions on when to progress to each step, thereby systematically navigating the clinical fluctuation that can occur in asthma.^51^ This image can be thought of (in the language of actor‐network theory) as an immutable mobile that has become accepted as “the way things are” and helped to stabilize asthma management routines in UK primary care over many years.^55^ Some GPs commented that this image was printed out and fixed on the walls of their clinics.

Introduction of FeNO testing in the primary care setting would require major changes to current organizational routines: a more complicated diagnostic algorithm based on an unfamiliar and expensive new test, requiring longer appointments and a shift of some work from nurses to GPs. Because of rising GP workload and the scarcity of respiratory‐trained primary care nurses, our informants considered the new routines for implementing FeNO unlikely to happen.

There's a real problem, real shortage of respiratory nurses. I mean, there's very, very few of them. The training is getting more and more and more complicated. … A lot of GPs have devolved, certainly the COPD stuff, to respiratory nurses. And there are very few of them. So, there's a big hole in a very, very complicated area.

—Nonspecialist GP 1

In the context of shrinking resources and rising workloads, the implications of a less efficient work routine were prohibitive. In one practice, nurses had been given a free demonstration device by a manufacturer, but when the supply of sensors ran out, GPs and commissioners had refused to invest further in the product, leading to testing devices “sitting in a box on a shelf,” according to one primary care respiratory nurse.

In short, from a primary care perspective, FeNO's positioning in NG80 had all the hallmarks of an unwelcome disruptive innovation.^17^ NICE's response to complaints that the diagnostic algorithm did not fit with primary care was that “service delivery aspects of the recommendations are outside the remit of this guideline.”^43^ ^(p77)^

### Microlevel Findings: Human and Technological Actors

The vignette in Box 2 illustrates the habitus of a world‐leading clinician and research authority on asthma. On a practical level, he is highly skilled in using the FeNO device, and he is able to instruct the patient how to control her breathing by reading the dial and listening to the noise. He knows not to panic when it does not work the first time, and he makes efficient use of the time while it loads the score. He draws on a vast body of knowledge about FeNO as a biomarker and the nuances of managing patients with non‐eosinophilic asthma. By drawing intuitively on both the here‐and‐now FeNO result and his extensive wider knowledge, he arrives at what he considers a more accurate diagnosis and recommends a change in treatment, which he justifies confidently in a letter to the patient's GP. When addressing colleagues, he projects wider notions of clinical excellence and progress symbolized by FeNO testing and precision medicine. Using authoritative rhetoric, he claims that had this approach been used earlier, the problems of misdiagnosis and overtreatment (which he attributes to symptom‐based management in primary care) would have been averted.

The vignette in Box 3 illustrates a very different habitus. Primary health care has been defined as doing simple things well, for large numbers of people, few of whom feel ill.^69^ The nurse in this vignette spends her time seeing such patients and aligns her work with the established principles of chronic disease management in low‐risk settings: registration, recall, regular review, and education for self‐management.^69^ Through her experience in this setting, she knows that the step‐up, step‐down approach works well for most patients. She perceives the patient's deterioration as due to his lack of compliance with the BTS‐SIGN guideline, not to flaws in the guideline itself. Furthermore, just as the consultant in Box 2 is skilled at supporting an inexperienced patient in using the FeNO device, this nurse is equally skilled at supporting her patient to use a technically simpler peak flow meter and in distinguishing a genuinely low reading from a device malfunction.

A superficial essentialist analysis of the vignettes in Boxes 2 and 3 might suggest that they reflect state‐of‐the‐art and primitive approaches to asthma care, respectively. Respondents from secondary care backgrounds framed GP resistance to the NG80 guideline in behaviorist terms relating to ignorance and stubbornness. They considered that the “fuss” would subside with familiarity.

We all just took it on the chin really, and kind of said, “Well, in maybe a couple of years—you know, maybe 10 years’ time—people will be so used to doing it that they'll forget there was ever a fuss about it.”

—NICE guideline development group member 2

The depiction of primary care as ignorant and clinically backward can be—and was—challenged. While a minority of our GP informants cautiously endorsed the broader precision medicine promises around FeNO testing and viewed it as potentially useful in primary care, most did not. They rejected the basic problematization (that asthma management in primary care suffered from inaccurate diagnosis and overtreatment) and described the FeNO test as having been “mis‐sold.” They questioned its sensitivity and specificity in a primary care population and were unconvinced that its introduction would improve outcomes compared to the registration‐recall‐review‐educate approach based on the BTS‐SIGN guideline and its step‐up, step‐down treatment ladder. Drawing on their knowledge as generalists (that is, experts in dealing with uncertainty in an unselected population where the signal‐to‐noise ratio is weak^69^), they argued that although the FeNO test might make sense in tertiary care, it was inappropriate in primary care.

[Precision medicine] is—you know, it's the golden bullet for the individual disease. So the notion is fantastic. But with … conditions like hypertension or asthma, the majority of people don't need a precise workup. Certainly not necessarily in biological terms. … I mean, the chair of the [NICE] guideline process was the tertiary referral specialist, you know? So, somebody working at a hospital would get the difficult cases referred by hospitals. So, a very small proportion of people with asthma in general practice are referred to hospital at all. And a very small proportion of those are referred to a tertiary center because there are difficulties with the diagnosis. This means somebody who works at that level is seeing all the really, really knotty diagnostic problems.

—General practitioner with special interest in asthma

Many GP informants thus viewed the trend toward precision medicine for asthma as reflecting a drive toward molecular reductionism and away from primary care's traditional holistic approach. Furthermore, aligned with their wider dispositions toward the politics of the health care system (notably, progressive underfunding of primary care), many GPs viewed the introduction of “required” precision medicine testing as a manifestation of a creeping managerialism that stifles professional autonomy and puts alleged efficiency concerns above the interests of patients.^70^

While primary care practitioners were reluctant to introduce FeNO testing routinely, some used it eclectically. One experienced respiratory nurse, for example, was an early adopter of the test and established a local referral hub for complex patients, allowing other practices to access the benefits of the test in selected cases (and also generating income for her practice):

So, if the patient presented with a history that wasn't absolutely clear cut of asthma but sounded like it probably was, and you needed that extra bit of confirmation that this was most likely asthma, we would do a FeNO test. … It's not a cheap test, so you have to be using it appropriately

—Primary care respiratory nurse 2

This example illustrates how the NG80 guideline contains assumptions about how and by whom the FeNO test will be used (all patients in all primary care asthma clinics). This nurse's antiprogram,^37^ in which FeNO testing is used selectively and judiciously within a primary care special‐interest service, draws on both specialist and generalist expertise and offers a potential way of resolving the current stand‐off between the primary and secondary care camps.

In sum, the very different perspectives on NG80 and FeNO testing in primary and tertiary care reflect different values, different kinds of expertise, and accumulated experience with different populations. Interviewees from both sectors were highly knowledgeable about their own scope of practice and depicted actors in the other sector as lacking key knowledge. The NG80 guideline, and the FeNO test as positioned in the guideline, “configured the user” in that it contained unjustified assumptions about who would use it and when. One or two creative actors in primary care have begun to push back against these assumptions by using the test differently and in a way that could potentially provide a more stable alignment of humans, technologies, and wider incentives.

## Discussion

This paper has described a case study of a so‐far unsuccessful attempt to introduce the logic of precision medicine into UK primary care using a government‐endorsed national guideline as a vehicle. Through detailed ethnographic and documentary analysis, and informed by a performative analysis using TESST, we have shown how academic advocates of precision medicine went through three of Callon's four steps of translation: problematization (constructing asthma management in primary care as primitive and characterized by overdiagnosis and poor tailoring of treatment), interessement (engaging policymakers and, specifically, the NICE guideline development group in an effort to fix the problem), and enrollment (defining particular roles that would support and stabilize the routine use of FeNO technology in primary care). The final stage of translation—mobilization of primary care professionals to enact the vision—has not occurred, largely because of a powerful pushback from primary care professional bodies.

This case is illustrative of the tensions introduced in the introductory section: an essentialist perspective on precision medicine–informed policy and practice—that is, a more or less inevitable development, but progress “slowed down” by scientific ignorance and bureaucratic reality—versus a performative perspective—that is, a questionable direction of travel, impelled by vested interests from academia and industry; an essentialist perspective on translation, in which innovations drive productive change, versus a performative perspective, in which complex struggles between interest groups may generate and legitimize innovations—or, alternatively, suppress and delegitimize them; and an essentialist perspective on clinical guidelines, as neutral conduits for scientific truth, versus a performative perspective, as “trailblazer” instruments for shaping what counts as truth. These tensions help explain how the story has unfolded so far.

At the macro level, three main social institutions are evident: the government and its agencies (what Scott would call the “regulative” institutional pillar), professional standards (Scott's “normative” pillar), and established traditions and practices (Scott's “cultural‐cognitive” pillar). Each pillar offers a different rationale for legitimacy, by virtue of being—respectively—legally mandated (what *must* happen), morally authorized (what *should* happen), or culturally sanctioned (what generally *does* happen). A small but influential group of professionals, academic respiratory physicians, managed to align with the government agency NICE and use what Scott calls coercive mechanisms in pursuit of their translational goal. *Normative* mechanisms, for example, conveying new standards of clinical excellence to other professionals, as when the consultant dictates a letter to the GP (Box 2), are also evident. Indeed, the pointed refusal of the Royal College of General Practitioners and the Primary Care Respiratory Society to endorse the NICE asthma guideline illustrates a clash between the regulative and normative pillars. There is no evidence of *mimetic* mechanisms, in which GP practices would seek to copy a precision medicine asthma service that was up and running elsewhere.

For a technology to be widely adopted, translation efforts have to overcome various challenges—what Latour calls “trials of strength”^55^—in the adopting system. The precision medicine academics’ translational effort failed not just because the regulatory pillar was relatively weak (NICE guidelines are not laws or must‐do regulations) and lacked financial incentives (indeed, at a time of worsening austerity and unprecedented workforce shortages, FeNO testing came with major financial disincentives), but also because the dominant professional pillar (eg, clinical excellence as defined by the British Medical Association, Royal College of GPs, Primary Care Respiratory Society, and BTS‐SIGN guidelines) and the cultural‐cognitive pillar (eg, the infrastructure of chronic disease management in primary care, with its decades‐old tradition of registration‐recall‐review‐educate delivered almost entirely in nurse‐led clinics) were relatively strong.

At the meso level of the GP practice, the prevailing institutional logic favored a script of continuing the business‐as‐usual model of nurse‐led asthma clinics and following the professionally endorsed BTS‐SIGN guideline rather than the government‐produced NICE guideline. At the micro level of clinical action, GPs and nurses had internalized these logics and experienced little or no cognitive dissonance as they continued their traditional approach.

What of the future of FeNO testing in primary care? Our data suggest that, as with many new technologies, “upgrades” may well emerge that alter the balance between benefits and disbenefits (hassles, costs, or harms). The device may, for example, become more dependable, easier to use, and cheaper. Other biomarkers that, combined with FeNO, would generate a full precision medicine patient profile may be developed. These and other advances, especially if undertaken in dialogue with primary care professionals, may clarify the place of biomarkers vis‐à‐vis traditional assessment in a low‐risk population. Perhaps, for example, asthma specialists might seek to join forces with a largely primary‐care‐based professional movement, Choosing Wisely, which aims to reduce under‐ and overdiagnosis and under‐ and overtreatment.^71^ There is also potential to enroll primary‐care‐based “super‐users” of FeNO devices (like the nurse described in the previous section) to provide a source of expertise and a “beacon” service that other providers may wish to replicate.

Through incremental change, then, precision medicine for asthma may achieve a foothold in primary care in the future. Our view is that it is unlikely to do so purely through the “Trojan horse” of a revised NICE guideline, for several reasons. First, the imbalance in the different institutional pillars described previously is likely to persist, meaning that precision medicine will not become the dominant discourse in primary care anytime soon. Second, practical realities will continue to trump deterministic visions for the FeNO technology. In our case study, members of the guideline development group and other precision medicine champions appeared to expect primary care to mold itself to the technology's “logical pattern”^72^ and underestimated the arduous work required to mobilize changes in a complex and resource‐stretched clinical setting. Third, the “gatekeeping” role of guidelines is likely to continue to dominate over the “trailblazing” role. Guideline production usually follows highly circumscribed bureaucratic rules, which constrain actors’ ability to project their preferred visions for change, so they tend to reach for compromises.^73^

Finally, hotly tipped biomarkers are unlikely to become practice as usual in primary care until they are validated in primary care populations, a step that has not yet happened with FeNO.^74^ In such circumstances, the “trailblazing” role of clinical guidelines will likely be eclipsed by their quality control functions, diminishing even further the prospect of a guideline bringing about “disruptive” changes to clinical practice.

Our findings resonate with other empirical case studies that have shown that precision medicine more generally faces a number of complex challenges in becoming widely adopted and mainstream in health care systems,^9^, ^75^, ^76^ particularly in the generalist domain of primary care.^77^ They suggest that primary care's resistance to the tools and classifications of precision medicine is attributable to more than a lack of organizational readiness. This resistance also stems from humanistic‐informed claims that primary care has always been a personalized, holistic practice, as well as from challenges by scientific medicine–oriented primary care physicians that such interventions are not as evidence‐based in this setting as their protagonists claim.^78^

The use of a single case study raises questions about the extent to which our findings are generalizable. Yin has argued that *theoretical* generalizability can be achieved only with “small‐N” samples in which each case is theoretically sampled in advance to test a specific instance of the theoretical phenomenon being investigated; theoretical insights are achieved through cross‐case comparison.^79^ Stake, in contrast, talks of *naturalistic* generalization, in which a detailed interpretive analysis of a single case can produce valuable insights about how the world works.^80^ The former approach relies heavily on abstraction, in which real‐world phenomena are expressed as variables, such as “entrepreneurship,” “innovation,” and “resistance”; the latter is more interested in what Shotter and Tsoukas have called “a living world of responsive relations”—for example, stories about particular entrepreneurs promoting particular innovations and meeting particular kinds of resistance.^81^ ^(p320)^ Tsoukas has called this approach heuristic generalization,^82^ an approach that depends on “theory's ineradicable dependence on the dynamics of the life‐world within which it has its ‘currency.’”^81^ ^(p311)^ In short, we believe the case described in this paper has produced important heuristic insights; we do not claim that our findings describe a pattern that will prove universal or invariable.

In conclusion, and with the caveat in the previous paragraph, our case study suggests that “radical” guidelines may succeed as catalysts of change only in a limited way for interested individuals and groups—for instance, by generating attention and interest toward a new technology (eg, FeNO) and linked ideas (eg, precision medicine). But in the absence of a wider program of professionally led and adequately resourced change efforts, the recommendations in such guidelines will lack meaning, legitimacy, and authority among intended users and will generally be strongly resisted.
